# Fixed Prosthetic Restorations and Periodontal Health: From Fabrication to Supracrestal Tissue Attachment Preservation and Clinical Outcomes

**DOI:** 10.3390/medicina62081454

**Published:** 2026-07-27

**Authors:** Marko Igić, Nadica S. Đorđević, Marija Đorđević, Aleksandra S. Milovanović, Nikola Gligorijević, Milica Kostić, Jana Pešić Stanković, Rodoljub Jovanović, Jelena T. Todić, Milena M. Kostić

**Affiliations:** 1 Clinic for Dental Medicine, Faculty of Medicine, University of Niš, 18000 Niš, Serbia; marko.igic@medfak.ni.ac.rs (M.I.); marija.jovanovic@medfak.ni.ac.rs (M.Đ.); nikola.gligorijevic@medfak.ni.ac.rs (N.G.); milena.kostic@medfak.ni.ac.rs (M.M.K.); 2 Department of Dentistry, Faculty of Medicine, University of Priština in Kosovska Mitrovica, 38220 Kosovska Mitrovica, Serbia; jelena.todic@med.pr.ac.rs; 3 Faculty of Medicine, University of Niš, 18000 Niš, Serbia; aleksandra.milovanovic98@gmail.com (A.S.M.); mimi.kostic0@gmail.com (M.K.); rodoljubj97@gmail.com (R.J.); 4 Public Health Institute Niš, Faculty of Medicine, University of Niš, 18000 Niš, Serbia; jana.pesic.stankovic@medfak.ni.ac.rs

**Keywords:** fixed prosthetic restorations, supracrestal tissue attachment, periodontium, biomechanical stability

## Abstract

*Background and Objectives*: Periodontal health represents a condition characterized by homeostasis between the teeth and the surrounding supporting tissues and implies the absence of inflammation, confirmed by the lack of bleeding, epithelial attachment loss, mobility, or periodontal pocket formation. Fixed prosthetic restorations may compromise periodontal health already during the fabrication phase, given that decisions made during their planning and placement affect long-term clinical outcomes. The aim of this manuscript was to provide insight into the interaction between periodontal tissues and fixed prosthetic restorations. *Materials and Methods*: A narrative literature review was conducted using a structured search of the PubMed/MEDLINE, Scopus, and Google Scholar databases to identify studies addressing periodontal health in relation to fixed dental prostheses. The impacts of tooth preparation, gingival retraction methods, supracrestal tissue attachment, periodontal phenotype, crown emergence profile, and marginal fit of various biomaterials were analyzed. *Results*: According to the literature data, subgingival margin placement and violation of the supracrestal tissue attachment (biological width) induce chronic inflammation, loss of epithelial attachment, and alveolar bone resorption. Gingival retraction agents, used in chemomechanical retraction procedures, particularly ferric sulfate-based agents, may lead to a transient but significant spike in inflammatory markers and dysbiosis of the subgingival microbiome. Contemporary approaches, such as the biologically oriented preparation technique and the use of CAD/CAM (Computer-Aided Design and Computer-Aided Manufacturing) technology, may improve the precision of marginal fit. Zirconia restorations exhibit significantly better biocompatibility and reduced microbial adhesion compared to metal–ceramic restorations. *Conclusions*: Biological response of the periodontium to fixed prosthetic restorations depends on the complex interaction between the material, position of the marginal line, and oral hygiene control. Regardless of the material used or the fabrication technique employed, adequate plaque control and regular maintenance remain key factors in preserving periodontal health.

## 1. Introduction

The increasing prevalence of fixed prosthetic restorations, crowns, and bridges further emphasizes the importance of a thorough understanding of their interaction with periodontal tissues. The term periodontium encompasses a group of anatomical structures that play a crucial role in supporting the teeth, ensuring their vascularization and proprioception, as well as protecting them from pathogenic oral biofilms. Periodontal health is of key importance for the longevity of prosthetic reconstructions. Attention directed toward specific laboratory and clinical procedures can help reduce the risk of periodontal diseases that occur after the placement of fixed restorations [1].

Periodontal health represents a condition characterized by homeostasis between the teeth and the surrounding supporting tissues and implies the absence of inflammation, confirmed by the lack of bleeding, epithelial attachment loss, mobility, or periodontal pocket formation. According to various studies, there is a relationship between periodontal health status and the longevity and retention of fixed prosthetic restorations. The health of periodontal tissues directly affects tooth durability; therefore, fixed prosthetic restorations, which may influence the condition of the periodontium, indirectly affect the duration of their clinical functionality as well [2,3,4].

Fixed prosthetic restorations may compromise periodontal health already during the fabrication phase, given that decisions made during their planning and placement affect long-term clinical outcomes. Several laboratory and clinical factors influencing the condition of the periodontium in the presence of prosthetic restorations have been identified [5,6,7]. Based on the literature data, preservation of the supracrestal tissue attachment (previously referred to as the biologic width) has been established as a key, unifying concept in the evaluation of these still insufficiently clarified interactions. Failure to respect this important dimension leads to impairment of periodontal health, initiating inflammatory processes, damage to the epithelial attachment, and, in advanced cases, progressive tooth mobility and ultimately tooth loss [8,9,10]. The supracrestal tissue attachment is defined as the distance from the epithelial attachment to the crest of the alveolar bone. Its violation may occur as a result of excessive tooth preparation, placement of the crown margin at or apical to the alveolar bone crest, or excessive reduction of occlusal surfaces. The result of all these factors is loss of epithelial attachment and development of periodontal disease [11].

Understanding the role of fixed prosthetic restorations in preserving periodontal health requires knowledge of numerous relevant terms and concepts. The biomechanical stability of fixed prosthetic restorations is determined by factors such as the precision of marginal fit and the biocompatibility of the materials used, as well as the design of the preparation and the restoration itself. Each of these factors may affect the functional longevity of the restoration and its ability to provide adequate marginal sealing [12].

The aim of this narrative review was to provide insight into the interaction between periodontal tissues and fixed prosthetic restorations based on a selected review of the literature from relevant electronic databases (PubMed/Medline, Scopus, and Google Scholar).

## 2. Materials and Methods

A comprehensive literature search was conducted to identify studies addressing periodontal health in relation to fixed dental prostheses. The search was performed using the following keywords: “tooth preparation”, “retraction methods”, “dental ceramics”, “metal-ceramics”, “marginal fit”, “crown”, “fixed dental prostheses”, “periodontium”, “marginal gap”, and “complications”.

Electronic databases, including PubMed/MEDLINE, Scopus, and Google Scholar, were systematically searched. The literature published from 1971 to May 2026 was searched and analyzed.

The inclusion criteria comprised original clinical studies (randomized controlled trials, prospective and retrospective cohort studies, case–control studies, and cross-sectional studies), systematic reviews, and meta-analyses evaluating the relationship between fixed dental prostheses and periodontal tissues. Studies investigating the influence of tooth preparation, gingival retraction methods, restorative materials, marginal adaptation, crown contours, finish line design, and prosthesis-related biological complications on periodontal health were also included. Only full-text articles published in the English language were considered.

The exclusion criteria included case reports, case series with a limited number of patients, conference abstracts, letters to the editor, editorials, studies not directly related to periodontal tissues and fixed prosthodontics, articles published in languages other than English, and studies with insufficient methodological or outcome data.

A total of 127 publications were screened. Studies meeting the inclusion criteria were included and critically analyzed. The restriction to English-language publications represents a limitation of this review, as relevant studies published in other languages may have been excluded, introducing the potential for language bias. Table 1 summarizes the study types included in this review.

This study was designed as a narrative review because it addresses multiple interrelated aspects of fixed prosthodontics and periodontal health, including tooth preparation, gingival retraction, restorative materials, marginal adaptation, finish line design, and biological complications. The available evidence is methodologically heterogeneous with respect to study design, populations, interventions, and outcome measures, precluding a meaningful systematic review or meta-analysis. A narrative approach was, therefore, considered more appropriate, as it enables a comprehensive synthesis of the available evidence, reflects the historical development of the field over more than five decades, and provides clinically relevant conclusions.

The main potential sources of bias in this narrative review include selection bias, language bias resulting from the inclusion of English-language publications only, publication bias, and the methodological heterogeneity of the included studies. Factors found to affect the periodontium include the following: iatrogenic damage during tooth preparation and impression, subgingival position of the finish line of the restoration, gingival retraction procedures, supracrestal tissue attachment, periodontal phenotype, emergence profile, and characteristics of dental materials.

## 3. Discussion of Factors Affecting Periodontal Health with Fixed Restorations

### 3.1. Tooth Preparation and Impression

The process of fabricating fixed prosthetic restorations plays a key role in determining clinical success and preserving periodontal health after the integration of the final restoration into the orofacial system.

The etiology of periodontal tissue damage during the fabrication of fixed restorations is multifactorial and includes various clinical and technical aspects of therapy, including tooth preparation and the position of the preparation margin [13,63,64], gingival retraction and impression-taking procedures [14,65,66,67], the fabrication and use of provisional restorations [14], the use of different types of cements, and the cementation procedure itself [100].

Periodontal diseases are characterized by a chronic inflammatory course and arise as a consequence of the body’s response to the presence of pathogenic microorganisms. Tooth preparation for fixed prosthetic restorations may cause mechanical damage to the gingival tissue, which is often accompanied by exudation and edema, thereby creating a favorable environment for the colonization and penetration of microorganisms. If not diagnosed in a timely manner and adequately treated, even mild gingival inflammation may progress toward the destruction of deeper periodontal structures, with permanent consequences for oral health, including tooth loss. The course and outcome of these diseases are largely determined by the immune response of the host [4,101].

Initial changes in the periodontal tissue, primarily in the form of gingival inflammation that subsequently leads to further destruction of periodontal tissues, may occur during tooth preparation [1,15]. The very process of removing hard dental tissue in order to create space for an artificial restoration is highly invasive and, in addition to causing reversible hyperemia of the dental pulp, also leads to changes in the gingival tissue, manifested by increased gingival index and bleeding index values [5].

The positioning of the finish line during tooth preparation represents one of the key determinants of the success of fixed prosthodontic therapy, particularly from the standpoint of preserving periodontal health and ensuring the longevity of prosthetic restorations. An adequately positioned preparation margin enables the preservation of the supracrestal tissue attachment and maintenance of biological balance in the periodontium, thereby reducing the risk of inflammatory changes and attachment loss.

In contrast, subgingival positioning of the finish line may lead to chronic gingival irritation, inflammation, infection, progressive attachment loss, and alveolar bone resorption [16,102]. Supragingival or controlled shallow subgingival placement of the preparation margin allows for better dental plaque control, facilitates oral hygiene maintenance, and reduces the likelihood of periodontal complications. The concept of extending into the gingival sulcus to a depth of up to 0.5 mm is also referred to as the “equigingival finish line” and is recommended for adequate retention and resistance of the artificial crown, as well as its esthetics [121].

Iatrogenic damage during the fabrication of fixed prosthetic restorations may compromise the supracrestal tissue attachment and predispose to the development of subgingival caries, as well as lead to uncontrolled inflammatory processes and destruction of periodontal tissues [122].

Nevins and Skurow emphasized that, in cases where subgingival positioning of the finish line is indicated, the dentist should avoid damaging the epithelial and connective tissue attachment during tooth preparation and impression taking. They also recommended limiting the extension of the subgingival margin to 0.5–1.0 mm, given that it is clinically impossible to precisely determine the boundary between the sulcular and junctional epithelium [68].

Studies conducted by Jovanović et al. on a homogeneous group of subjects with periodontally treated teeth demonstrated an increase in gingival index and bleeding index values immediately after subgingival tooth preparation. The values of these indices remained elevated compared to baseline at both 24 and 72 h after preparation, but gradually decreased over time, indicating the reversibility of the process. In addition, cytomorphometric measurements showed an increase in inflammatory markers, with a tendency for their values to decline over time. The reversibility of these changes suggests that adherence to the principles of tooth preparation can prevent permanent damage to periodontal tissues [5].

The same group of authors studied the increase in matrix metalloproteinase-9 (MMP-9), as an inflammation marker in periodontal diseases, before and after the preparation of a previously restored homogeneous group of teeth. They showed that the values of this proinflammatory cytokine increase, but also that MMP-9 levels return to the baseline range over time (after three days), indicating the absence of permanent gingival damage during tooth preparation. Lower values were observed in the case of an equigingival compared to a subgingival finish line position. The same study also suggested a tendency for an increase in the number of anaerobic microorganisms in the gingival crevicular fluid. The results revealed a more pronounced increase in *Aggregatibacter actinomycetemcomitans* in association with a subgingivally positioned finish line, whereas equigingival margins were associated with less pronounced alterations in the oral microbiota. In addition, a decrease in *Prevotella intermedia* and *Tannerella forsythia* was observed following preparation, particularly in the group with equigingival margins. These findings suggest a greater periodontal risk associated with subgingival finish lines and the need for further research into their impact on gingival health [17].

Infectious periodontopathogens represent a complex group of predominantly anaerobic bacteria within the subgingival biofilm, among which *Porphyromonas gingivalis*, *Tannerella forsythia*, and *Treponema denticola* (the so-called “red complex”), as well as *Aggregatibacter actinomycetemcomitans*, *Fusobacterium nucleatum*, and *Prevotella intermedia*, are particularly prominent. These microorganisms play a key role in the initiation and progression of periodontal diseases through the production of virulence factors and modulation of the host immune response, ultimately leading to chronic inflammation, connective tissue degradation, and alveolar bone resorption [18,69,123]. The contemporary concept of periodontitis pathogenesis is based on oral microbiome dysbiosis, whereby the dominance of periodontopathogenic species disrupts homeostasis and triggers destructive immunoinflammatory processes in periodontal tissues [124].

Damage to the periodontal tissue during impression-taking is associated with the gingival retraction procedure, which aims to achieve apical and lateral displacement of the marginal gingiva and dry the gingival sulcus area to obtain a more accurate impression of the finish line. Optimal impression-taking of this demarcation zone is equally important in digital impression procedures as well. The most commonly employed method is the mechanical–chemical technique, which involves the use of a retraction cord impregnated with either an astringent agent (aluminum or iron salts) or a vasoconstrictor (epinephrine) [19]. However, the insertion of the retraction cord into the gingival sulcus may result in mechanical or chemical damage to the junctional epithelium, thereby creating conditions favorable for the development of inflammation that may progress into periodontal disease. Therefore, careful selection of cord thickness according to the gingival phenotype and sulcus depth is essential, as well as the limitation of the duration of the retraction agent action to only a few minutes [118].

The literature data indicate that astringent agents, which exert their effects through protein precipitation and inhibition of transcapillary plasma protein passage, demonstrate a certain degree of aggressiveness that may result in tissue damage during gingival retraction procedures [70,119]. The results of these studies indicate the existence of cytotoxic and inflammatory effects of these compounds [7,103]. The literature findings emphasize the importance of clinical studies that utilize objective gingival indices and quantification of proinflammatory cytokines to enable precise assessment of gingival tissue response following the application of retraction cords [70,119]. In this context, gingivitis is characterized as a reversible inflammatory condition induced by the accumulation of bacterial biofilm, whereby bacterial products activate immune system cells and induce the release of inflammatory mediators, including interleukin-1 (IL-1), interleukin-6 (IL-6), tumor necrosis factor-alpha (TNF-α), and matrix metalloproteinase (MMP) [20].

A prospective clinical study conducted by Igić et al. involving 60 subjects evaluated the effects of chemical–mechanical gingival retraction on the gingival index (GI) and salivary concentrations of IL-6 and TNF-α using retraction agents based on aluminum chloride and ferric sulfate. The results demonstrated a statistically significant increase in all parameters 24 h after the intervention, followed by partial reduction after 72 h, yet still above the initial values. These findings indicate transient, moderate gingival inflammation following retraction, more pronounced when associated with ferric sulfate-based agents [6].

These findings are consistent with another study by the same group of authors that evaluated the gingival bleeding index (GBI) and salivary concentrations of monocyte chemoattractant protein-1 (MCP-1). That study likewise reported a significant increase in inflammatory parameters 24 h after gingival retraction, particularly in prepared teeth and in cases where ferric sulfate agents were used, followed by partial regression after 72 h. Together, these results confirm that chemical–mechanical gingival retraction induces a transient yet clinically significant inflammatory response of the gingival tissue [71].

### 3.2. Supracrestal Tissue Attachment

The supracrestal tissue attachment, traditionally referred to as the biologic width, represents a dynamic morphofunctional unit of the periodontium consisting of the junctional epithelium and the supracrestal connective tissue attachment [21]. Its integrity is crucial for the maintenance of periodontal health, given that the disruption of this space activates adaptive, and often destructive, biological processes aimed at reestablishing the physiological dimension of the attachment, consequently positioned more apically. The dimensions of the supracrestal tissue attachment represent the sum of the vertical components located between the crest of the alveolar bone and the free gingival margin. Classical histological studies indicate that this space consists of approximately 1.0 mm of connective tissue attachment and about 0.97 mm of junctional epithelium, which in total amounts to approximately 2.0 mm of supracrestal dimension [72].

In the mid-20th century, Gargiulo et al. described the “biologic width” as consisting of the following components: a sulcus depth of 0.69 mm, junctional epithelium measuring 0.97 mm (0.71–1.35 mm), and supra-alveolar connective tissue attachment measuring 1.07 mm (1.06–1.08 mm). Based on these, the total biologic width is commonly reported as 2.04 mm, which represents the sum of the epithelial and connective tissue components [73].

However, contemporary research emphasizes that these values are not absolute, and that they demonstrate substantial individual variability depending on gingival biotype, tooth position, patient age, and local periodontal conditions. When sulcus depth, averaging approximately 0.5–1.0 mm, is also taken into consideration, the total dimension from the crestal bone to the gingival margin may reach approximately 2.5 to 3.0 mm [22].

The clinical significance of these dimensions is reflected in planning restorative margin placement, where preservation of at least 3 mm between the crestal bone and the finish line of the preparation is recommended to prevent disruption of the supracrestal tissue attachment and the subsequent development of periodontal complications [21,23,24,25,26,27]. In this context, fixed prosthodontic procedures, particularly those involving subgingival margin placement, may contribute to periodontal tissue alterations when biological principles are not respected. Therefore, the position of the restoration margin should be determined according to the gingival phenotype, with subgingival placement limited to approximately 0.3–0.5 mm in thin phenotypes and up to 1.0 mm in thick phenotypes, provided that the supracrestal tissue attachment is preserved [22,24]. In patients with periodontal disease, subgingival margins should be avoided whenever clinically feasible, as they may promote plaque accumulation, impair plaque control, exacerbate periodontal inflammation, and increase the risk of further attachment loss [23,24].

Histological and clinical data indicate that the invasion of restorative margins into the supracrestal tissue attachment results in apical migration of the junctional epithelium and reorganization of the connective tissue, which is often accompanied by resorption of the crestal alveolar bone. This process represents a biological attempt of the organism to reestablish an appropriate dimension of the supracrestal space. Similar adaptive mechanisms have also been observed following complete removal of the connective tissue attachment during tooth preparation or surgical procedures, where the formation of a new attachment occurs at a more apical position relative to the original site, accompanied by simultaneous bone remodeling. It is important to point out that temporary detachment of the junctional epithelium does not necessarily result in permanent damage, given its capacity for reattachment in areas not covered by restorative material [74,75].

The response of periodontal tissues, however, depends not only on the anatomical position of the crown margin but also on the characteristics of the restorative material. These findings highlight the importance of the physicochemical properties of restorative materials in modulating tissue response, including surface roughness, chemical stability, and adhesive capacity [28,29].

Considering the above, preservation of the supracrestal tissue attachment may be regarded as one of the fundamental principles of contemporary restorative and prosthodontic therapy. Treatment planning should, therefore, be based on precise assessment of the relationship between restoration margins and periodontal structures, together with careful selection of materials and techniques, in order to minimize the risk of inflammation, attachment loss, and bone resorption, and to ensure long-term functional and esthetic success of therapy [21,29,30,31,32,76,77].

The study results further indicate that lingual tooth surfaces exhibit a greater tendency for plaque accumulation compared to vestibular surfaces, which may have significant implications for periodontal tissue health. In addition, the risk of gingival bleeding is approximately twofold higher when the margins of posterior crowns are placed subgingivally compared with supragingival margin placement [78]. The position of restorative margins within the junctional epithelium and supracrestal connective tissue attachment may lead to gingival inflammation and potential gingival recession. Nevertheless, the presence of finish lines within the gingival sulcus does not necessarily result in gingivitis, provided that patients maintain adequate oral hygiene and undergo regular professional check-ups [33].

### 3.3. Gingival Phenotype

One of the key factors influencing the relationship between fixed prosthodontic restorations and periodontal tissue health is the periodontal phenotype, which includes gingival thickness, keratinized tissue width, and buccal bone plate thickness. Unfavorable characteristics of this phenotype increase susceptibility to recession [24,79].

Inadequate treatment planning, particularly when essential clinical factors such as gingival phenotype are disregarded, may lead to mucogingival conditions associated with the development of gingival recession, characterized by apical displacement of the gingival margin accompanied by attachment loss [24].

Failure to consider the gingival phenotype during fixed prosthodontic treatment planning may negatively affect the existing periodontal condition. Therefore, gingival sulcus depth, gingival thickness, and the position of the alveolar crest, which exhibit individual variability, must be taken into consideration during therapy [34,35,80,81]. Gingival thickness represents a key component of its phenotype, given that thicker tissues exhibit greater resistance to mechanical and inflammatory impacts, whereas thin tissues are more susceptible to recession and attachment loss. The gingival phenotype significantly influences treatment outcomes, and inadequate tooth preparation together with violation of the biologic width may result in progressive thinning of the tissue, such that even an initially thick phenotype may transition into a thin phenotype because of prosthodontic therapy [36,82]. A study by León-Martínez et al. [37] demonstrated that teeth prepared with horizontal finish lines (chamfer or shoulder) have less favorable periodontal parameters compared to unprepared control teeth, including greater attachment loss and gingival migration.

Contemporary approaches, such as the biologically oriented preparation technique (BOPT), enable reaching a more stable relationship between soft tissues and a prosthetic restoration using feather-edge preparation without flap elevation, with favorable esthetic and functional results [38,39]. Agustín-Panadero et al. [83] studied the clinical performance of crowns and fixed partial restorations fabricated using vertical preparation without a defined finish line (BOPT). After a two-year follow-up period, gingival thickening, marginal tissue stability, and satisfactory esthetic results were observed, with no mechanical complications associated with the restorations.

Even though periodontal health may be maintained regardless of the phenotype, certain clinical parameters, such as the gingival index, may deteriorate in the presence of subgingivally positioned preparation margins, particularly when the width of the keratinized tissue is less than 2 mm [79]. In patients with a limited zone of keratinized gingiva, it is advisable to avoid deep subgingival placement of the finish line and emphasize the significance of oral hygiene and frequent check-ups.

### 3.4. Crown Emergence Profile

The emergence profile refers to the shape and contour of the cervical portion of a tooth or restoration in the transition zone from the subgingival to the supragingival region, i.e., the manner in which the restoration “emerges” from the gingival tissue [40]. In a narrower sense, the emergence profile refers exclusively to the morphology of the part of the restoration that is in direct relationship with the gingival sulcus and marginal gingiva, encompassing several millimeters above and below the gingival margin. Unlike the overall crown convexity (height of contour), the emergence profile describes the fine cervical contour that directly influences the relationship between the restoration and soft tissues, as well as accessibility for oral hygiene maintenance [41,42,43].

Its design has a significant influence on the health of the periodontium, as it determines the conditions for plaque accumulation and the response of the gingival tissue [44]. An excessively convex emergence profile may impair oral hygiene and contribute to the development of inflammation, whereas an insufficiently contoured profile may compromise gingival tissue support [43,45,125,126]. Experimental studies indicate that increased convexity may be associated with a more pronounced inflammatory response [46], although some authors have reported no significant differences in their studies [120].

Clinical studies in humans have demonstrated that pronounced overcontouring of restorations may lead to an increase in the gingival index values and plaque accumulation [84]. Nevertheless, these findings cannot be directly applicable to the emergence profile in the narrower sense [85]. Conversely, studies involving smaller sample sizes suggest that minor variations in restoration contour (0.5–1 mm) do not significantly affect periodontal parameters, provided that adequate oral hygiene is maintained [86,87].

Therefore, a properly designed emergence profile that follows the natural tooth anatomy and enables effective plaque control represents an important factor in preserving periodontal health and ensuring the long-term success of restorative therapy.

Gingival morphology largely depends on the shape and anatomy of teeth. Square-shaped teeth are associated with more favorable esthetic outcomes due to longer approximal contact areas and a less pronounced papillary space, whereas a triangular tooth shape is characterized by more incisally positioned approximal contact, which requires greater papillary height to fill the interdental space. Such morphology increases the risk of gingival recession and the appearance of so-called “black triangles” [88,89].

### 3.5. Marginal Fit of Crowns and Biometrical Selection

Optimal adaptation of the marginal edge of an artificial crown, i.e., the absence of discrepancy between the prosthetic restoration and the tooth, is essential not only for its structural rigidity but also for the preservation of pulpal and periodontal health [47]. Increased marginal discrepancy implies a thicker layer of cement, which is more susceptible to dissolution, as well as enhanced dental plaque accumulation and microleakage. Consequently, marginal discoloration, increased gingival crevicular fluid flow, recurrent caries, pulpal infection, periodontal lesions, and alveolar bone resorption may occur, ultimately leading to prosthetic treatment failure [47] (Table 2).

There is no universal consensus regarding the optimal marginal gap value. Some authors consider values below 120 μm to represent an acceptable clinical standard [104,105,106], whereas others advocate stricter criteria below 100 μm [107,108]. At the same time, the literature suggests ideal values should range between 20 and 75 μm [47].

Particular importance is attributed to the localization of the marginal edge, i.e., its relationship to the gingival margin (supragingival, equigingival, or subgingival position). Supragingival margins enable adequate oral hygiene and are associated with a lower incidence of secondary caries and periodontal diseases. In contrast, subgingivally positioned crown margins hinder oral hygiene maintenance and require careful clinical assessment due to the increased risk of inflammatory changes [48].

CAD/CAM (Computer-Aided Design/Computer-Aided Manufacturing) restorations demonstrate a tendency toward better marginal adaptation [109] and often achieve potentially better precision compared to heat-pressed lithium disilicate ceramic restorations [110]. However, some studies failed to identify statistically significant differences compared to conventional fabrication techniques [111]. Compared to metal–ceramic restorations, all-ceramic CAD/CAM restorations generally exhibit greater precision [112], particularly when digital impression techniques are employed [113]. Furthermore, these restorations demonstrate better overall adaptation compared to conventionally fabricated restorations [114], whereas zirconia copings maintain structural stability at marginal areas without phase transformations, regardless of preparation geometry [115].

Clinical studies indicate that poor oral hygiene occurs in a significant proportion of patients one year after insertion, regardless of the type of prosthetic restoration used (metal–ceramic, glass–ceramic, or zirconia–ceramic) [103]. At the same time, fixed prosthetic restorations may compromise host defense mechanisms by creating retentive surfaces favorable for biofilm accumulation, which may lead to periodontal tissue damage [90].

The study by Avetisyan et al. [1] showed that zirconia–ceramic CAD/CAM restorations provide more favorable periodontal outcomes and reduced inflammation compared to Co-Cr constructions.

The progression of inflammation leads to the development of periodontitis, characterized by irreversible attachment loss, alveolar bone resorption, and periodontal pocket formation [33,49,50,51]. Clinically, conventional metal–ceramic restorations are more frequently associated with gingival discoloration and gingivitis, whereas these signs are less commonly observed in CAD/CAM-fabricated restorations and are particularly absent in zirconia–ceramic restorations, which exhibit better biocompatibility and reduced microbial adhesion [52,91,107].

CAD/CAM restorations generally demonstrate a more favorable periodontal response compared to conventional techniques, even though their success is influenced by numerous factors, including biomaterial, fabrication technology, and conditions within the oral environment [53,92,116]. In the long term, zirconia–ceramic restorations are associated with more favorable periodontal parameters compared to metal–ceramic constructions, although differences in certain clinical indicators are not statistically significant [50].

In addition, the type of prosthetic restoration exerts a greater influence on gingival health than the biomaterial itself, with bridges more frequently associated with deterioration of oral hygiene and gingival indices compared to single crowns [93,94]. Regardless of the material used, adequate plaque control and oral hygiene remain key factors in the prevention of periodontal complications [95,96].

A study by Al-Sinaidi et al. [96] demonstrated that abutment teeth supporting fixed restorations exhibit poorer periodontal parameters compared to single crowns, especially in older patients and after a prolonged functional period.

Increased biofilm accumulation and inflammation require the implementation of both mechanical (toothbrushes, interdental cleaning aids) and chemical plaque-control measures. Among chemical agents, chlorhexidine (0.2%) has been shown to be the most effective, followed by essential oils, whereas cetylpyridinium chloride demonstrates favorable efficacy but also carries a greater risk of adverse reactions [54,97,98].

## 4. Discussion

Fixed prosthetic restorations play a significant role not only in prolonging the functional lifespan of dentition but also in improving esthetics. However, their impact on periodontal health represents an important clinical concern, given that long-term complications may lead to treatment failure. The diagnosis and monitoring of complications are essential steps in preserving periodontal health and preventing irreversible changes. Clinical assessment includes the measurement of periodontal pocket depth, tooth mobility evaluation, the assessment of bleeding on probing, and visual examination of gingival condition.

The contemporary understanding of the relationship between fixed prosthetic restorations and the periodontium requires critical evaluation of available evidence in the context of advances in materials and technologies. Earlier studies have limited applicability in modern clinical practice due to substantial improvements in biocompatibility and precision of contemporary ceramic materials and CAD/CAM systems [55].

The results indicate that the response of periodontal tissues largely depends on material characteristics, with modern adhesive and ceramic systems demonstrating a more favorable biological profile compared to traditional materials [117]. Nevertheless, marginal precision and the position of the finish line remain critical factors, given that subgingival margins may increase the risk of inflammation and plaque accumulation, even though this effect can be modified through adequate oral hygiene and proper restoration design. In this regard, the results of the present study are consistent with reports in the literature, confirming that less favorable periodontal parameters are more commonly associated with more invasive prosthetic approaches and subgingivally positioned marginal edges [56,99].

Despite certain inconsistencies in the literature, there is a clear consensus that the preservation of periodontal health around fixed restorations largely depends on plaque control and regular maintenance, rather than prosthetic factors exclusively [57,58]. Even optimally fabricated restorations may lead to complications under conditions of inadequate oral hygiene, which emphasizes the importance of patient education and motivation as integral components of the therapeutic approach. The inflammatory response of the gingiva may occur even in technically well-fabricated restorations if the conditions for adequate hygiene maintenance are not met.

Furthermore, individual periodontal characteristics, particularly the periodontal phenotype, play a significant role in treatment planning. The assessment of gingival thickness, keratinized tissue width, and alveolar bone morphology enables the optimization of prosthetic design and reduction of complication risk. Although the concept of supracrestal tissue attachment remains the cornerstone of contemporary prosthodontics, its universal application is a matter of debate due to the adaptive capacity of tissues and variability of clinical conditions. In clinical practice, margin placement and adherence to biological principles remain among the most important modifiable factors influencing the long-term outcome of therapy [34,59,60,61].

Advances in the development of materials and techniques, including biologically oriented preparation techniques, have shown promising results. However, their application remains limited by the lack of long-term clinical studies and standardized protocols [62]. Moreover, certain limitations of the available studies should be considered, including relatively small sample sizes, methodological heterogeneity, and reliance on indirect clinical parameters, all of which may affect the interpretation of the results and their generalization.

In this context, the results should be regarded both as a contribution to existing knowledge and as a basis for further research. Future studies should include larger sample sizes, longer follow-up periods, and standardized evaluation protocols in order to define risk factors more precisely and further improve clinical practice.

## 5. Conclusions

In conclusion, the findings of this review, consistent with contemporary evidence, indicate that the biological response of the periodontium to fixed prosthetic restorations depends on the complex interaction between restorative materials, marginal design and position, and effective plaque control. Subgingivally placed restoration margins and more invasive prosthodontic procedures are associated with less favorable periodontal outcomes, whereas contemporary ceramic materials and CAD/CAM technologies demonstrate superior biocompatibility.

Regardless of the restorative material or fabrication technique, effective plaque control and regular supportive care remain the key determinants of long-term periodontal health. Therefore, the long-term success of fixed prosthodontic therapy relies on an individualized treatment approach, adherence to biological principles, and active patient participation in maintaining optimal oral hygiene.

The methodological heterogeneity of the included studies and the absence of a formal risk-of-bias assessment limit the ability to draw definitive conclusions. Nevertheless, this narrative review provides a comprehensive synthesis of the available evidence and offers clinically relevant recommendations to support clinical decision-making in everyday dental practice.

## Figures and Tables

**Table 1:** Classification of studies included in the review.

Type of Study	References	Number of Studies Included in the Review
Review	[1,2,3,4,7,8,9,10,13,14,15,16,17,18,19,20,21,22,23,24,25,26,27,28,29,30,31,32,33,34,35,36,37,38,39,40,41,42,43,44,45,46,47,48,49,50,51,52,53,54,55,56,57,58,59,60,61,62]	59
Clinical study	[5,6,11,12,13,63,64,65,66,67,68,69,70,71,72,73,74,75,76,77,78,79,80,81,82,83,84,85,86,87,88,89,90,91,92,93,94,95,96,97,98,99]	42
In vitro study	[100,101,102,103,104,105,106,107,108,109,110,111,112,113,114,115,116,117]	18
Animal experiment	[118,119,120]	3

**Table 2:** Overview of conclusions and recommendations regarding marginal fit.

Study	Type of Article	Conclusions and Recommendations
Freire et al. [112]	original article	Zirconia restorations presented better marginal fit than metal–ceramic crowns.
Strimaneepong et al. [47]	review	Zirconium-based restorations made from computer-aided design provided better results than other materials.
Katarasli et al. [104]	original article	There are differences in marginal fit between different types of zirconia.
Felemban et al. [105]	review	Authors suggested potential benefits of supragingival position of crowns.
Reich et al. [107]	original article	IPS-Empress and Cerec 3D all-ceramic systems presented clinically acceptable gaps less than 100 microm.
Neves et al. [111]	original article	Lithium disilicate crowns fabricated by the Corect Bluem scanner exhibited a significantly smaller vertical misfit than crowns fabricated by a 3D laser scanner.

## Data Availability

No new data were generated during this study.
